# Evolving Epidemiological Characteristics of COVID-19 in Hong Kong From January to August 2020: Retrospective Study

**DOI:** 10.2196/26645

**Published:** 2021-04-16

**Authors:** Kin On Kwok, Wan In Wei, Ying Huang, Kai Man Kam, Emily Ying Yang Chan, Steven Riley, Ho Hin Henry Chan, David Shu Cheong Hui, Samuel Yeung Shan Wong, Eng Kiong Yeoh

**Affiliations:** 1 JC School of Public Health and Primary Care The Chinese University of Hong Kong Hong Kong China (Hong Kong); 2 Stanley Ho Centre for Emerging Infectious Diseases The Chinese University of Hong Kong Hong Kong China (Hong Kong); 3 Shenzhen Research Institute The Chinese University of Hong Kong Hong Kong China (Hong Kong); 4 GX Foundation Hong Kong China (Hong Kong); 5 School of Public Health Imperial College London London United Kingdom; 6 Department of Medicine and Therapeutics The Chinese University of Hong Kong Hong Kong China (Hong Kong); 7 Centre for Health Systems and Policy Research The Chinese University of Hong Kong Hong Kong China (Hong Kong)

**Keywords:** SARS-CoV-2, COVID-19, evolving epidemiology, containment delay, serial interval, Hong Kong, epidemiology, public health, transmission, China, intervention, case study

## Abstract

**Background:**

COVID-19 has plagued the globe, with multiple SARS-CoV-2 clusters hinting at its evolving epidemiology. Since the disease course is governed by important epidemiological parameters, including containment delays (time between symptom onset and mandatory isolation) and serial intervals (time between symptom onsets of infector-infectee pairs), understanding their temporal changes helps to guide interventions.

**Objective:**

This study aims to characterize the epidemiology of the first two epidemic waves of COVID-19 in Hong Kong by doing the following: (1) estimating the containment delays, serial intervals, effective reproductive number (R_t_), and proportion of asymptomatic cases; (2) identifying factors associated with the temporal changes of the containment delays and serial intervals; and (3) depicting COVID-19 transmission by age assortativity and types of social settings.

**Methods:**

We retrieved the official case series and the Apple mobility data of Hong Kong from January-August 2020. The empirical containment delays and serial intervals were fitted to theoretical distributions, and factors associated with their temporal changes were quantified in terms of percentage contribution (the percentage change in the predicted outcome from multivariable regression models relative to a predefined comparator). R_t_ was estimated with the best fitted distribution for serial intervals.

**Results:**

The two epidemic waves were characterized by imported cases and clusters of local cases, respectively. R_t_ peaked at 2.39 (wave 1) and 3.04 (wave 2). The proportion of asymptomatic cases decreased from 34.9% (0-9 years) to 12.9% (≥80 years). Log-normal distribution best fitted the 1574 containment delays (mean 5.18 [SD 3.04] days) and the 558 serial intervals (17 negative; mean 4.74 [SD 4.24] days). Containment delays decreased with involvement in a cluster (percentage contribution: 10.08%-20.73%) and case detection in the public health care sector (percentage contribution: 27.56%, 95% CI 22.52%-32.33%). Serial intervals decreased over time (6.70 days in wave 1 versus 4.35 days in wave 2) and with tertiary transmission or beyond (percentage contribution: –50.75% to –17.31%), but were lengthened by mobility (percentage contribution: 0.83%). Transmission within the same age band was high (18.1%). Households (69.9%) and social settings (20.3%) were where transmission commonly occurred.

**Conclusions:**

First, the factors associated with reduced containment delays suggested government-enacted interventions were useful for achieving outbreak control and should be further encouraged. Second, the shorter serial intervals associated with the composite mobility index calls for empirical surveys to disentangle the role of different contact dimensions in disease transmission. Third, the presymptomatic transmission and asymptomatic cases underscore the importance of remaining vigilant about COVID-19. Fourth, the time-varying epidemiological parameters suggest the need to incorporate their temporal variations when depicting the epidemic trajectory. Fifth, the high proportion of transmission events occurring within the same age group supports the ban on gatherings outside of households, and underscores the need for residence-centered preventive measures.

## Introduction

The novel coronavirus (SARS-CoV-2), which causes COVID-19, first appeared in Wuhan, China, in late December 2019 and quickly plagued the globe. The World Health Organization declared COVID-19 a pandemic on March 12, 2020. As of September 27, 2020, there have been 32.7 million cases and almost one million deaths worldwide [1]. Countries are experiencing the resurgence of COVID-19. For example, in the week of September 21-27, 2020, there were about 420,000 new cases in Europe [1], triggering another round of lockdown measures [2]. Researchers promptly summarized the case epidemiology during the early phase of the pandemic [3,4]. However, the enactment of nonpharmaceutical interventions and the presence of multiple genetic SARS-CoV-2 clusters [5] hint at important changes in the epidemiology of COVID-19.

Hong Kong is no exception with regard to COVID-19. The first epoch of the first wave of COVID-19 in Hong Kong took off after the first imported case reported on January 23, 2020 [6,7]. Initially, the epidemic was under control after prompt bundled public health interventions [8]. With the number of infections surging worldwide in mid-March 2020, Hong Kong faced the second epoch of the first wave of infection. Compulsory laboratory tests for all arriving passengers followed by 14-day compulsory quarantine spurred overseas residents to return, resulting in a small influx of imported cases. After peaking in March 2020, the number of cases remained low until a surge of local cases in July 2020, signifying the second wave of the epidemic in Hong Kong. This second wave represented the largest local outbreak in Hong Kong, which was likely attributable to initiation by imported cases coupled with the easing of social-distancing measures in July 2020.

The disease course of COVID-19 is governed by important epidemiological parameters, including containment delay and serial interval. The former has been shown to be associated with the infection source and number of doctor consultations [6], which in turn vary as the epidemic progresses, whereas the latter varies by virus type and subtype [9,10], the contact patterns between susceptible and infectious individuals [10], and the implementation of nonpharmaceutical interventions during epidemics [11].

Parameterization of mathematical models that account for the temporal variation of epidemiological characteristics would improve decisions regarding mitigation strategies. Moreover, containment delay increases opportunities for transmission and affects the effectiveness of control measures, whereas investigating ways to reduce containment delay could enhance control measures [12]. As such, we analyzed laboratory-confirmed COVID-19 case series in Hong Kong between January 2020 and August 2020 to quantify and identify the factors associated with the containment delay and serial interval.

## Methods

### Data Retrieval

We analyzed the case series provided by the Hong Kong Centre for Health Protection (HKCHP) from January 23, 2020, to August 2, 2020, from which we extracted the following: demographics, case classification, travel history, epidemiological links among cases, date of symptom onset, date of isolation, and the report date. Based on the order of settings embedded in a cluster and the case classification (cases, or close contact of cases), we compiled a line-list database of infector-infectee pairs (hereafter denoted as “paired data”).

### Definitions

A laboratory-confirmed case (hereafter denoted as “a case”) and a cluster were defined previously [6]. In short, a case refers to an individual with SARS-CoV-2 detected in a clinical specimen, and a cluster refers to at least two cases that are epidemiologically linked. Further, a local cluster is defined as a cluster that consists of at least one local case. A cluster encompasses one or more orders of settings, referred to as primary, secondary, tertiary, and quaternary settings.

Containment delay and serial interval were defined previously [6]. In short, containment delay is the time elapsed between the first onset of symptoms and mandatory isolation of a case [6], and serial interval is the time interval between the symptom onset of an infector and an infectee [13]. Further, secondary transmission refers to the first generation of infections induced by a case, and infections caused by infectees of a secondary transmission are referred to as tertiary transmission. Accordingly, subsequent orders of transmission are named based on the aforementioned rationale. Effective reproductive number (R_t_) is the average number of secondary cases generated by a primary case at any given time. It measures real-time transmissibility in response to control measures. The epidemic will shrink if R_t_ is consistently smaller than 1, and vice versa.

### Classifications of Cases

The HKCHP classified cases into six types according to their likely source of infection: imported cases, local cases, possibly local cases, and cases with epidemiological linkage with imported, local, or possibly local cases. Based on their travel history during the 14 days preceding the first symptom onset and their involvement in local clusters, the latter four types of cases were reclassified (Table 1) such that there were only three types of cases: (1) imported, (2) local, and (3) unclassified.

**Table 1:** Reclassification regime of cases.

Original classification	Reclassified classification	Number of cases (N=3512)
Imported cases	Imported cases	1045
Local cases	Local cases	901
Possibly local cases	Local cases^a^	2
Possibly local cases	Imported cases^b^	71
Possibly local cases	Unclassified cases^c^	30
Close contact of imported cases	Local	31
Close contact of local cases	Local	1370
Close contact of possibly local cases	Local	62

^a^Cases without travel history during the 14 days before their first symptom onset.

^b^Cases that had travel history during the 14 days before their first symptom onset and were not involved in any local cluster.

^c^Cases who either had travel history during the 14 days before their first symptom onset and were linked to a local cluster or did not have an onset/arrival date.

### Symptom Profile

Symptoms manifested by cases, if any, were grouped based on the International Statistical Classification of Diseases and Related Health Problems (ICD-10) and the national ambulatory medical care survey [14] into 8 categories (Table S1 in Multimedia Appendix 1), including general (such as fever and headache) and respiratory (such as cough and sore throat) symptoms.

### Mobility Index

The index is generated by Apple Maps, and represents the relative volume of routing requests (walking) for directions in Hong Kong compared to the baseline volume on January 13, 2020 [15]. The higher the index value is from the baseline, the higher the level of mobility. For ease of presentation, this index was further normalized with the value on January 18, 2020, set as 100.

### Statistical Analysis

Characteristics of cases and epidemiological parameters were summarized with mean, standard deviation, percentage, frequency, and bootstrapped confidence interval. Missing isolation dates were replaced by rounding up the mean of available isolation days in a stratum, which was made up of cases that had the same likely source of infection, asymptomatic indicator, quarantine status, mode of detection, and report dates. If there was no available isolation date in a stratum, the mean day differences between report dates and isolation dates were calculated, and the missing isolation dates were imputed as follows: the corresponding report dates minus the mean day difference. For containment delay, only local cases who were neither quarantined nor under medical surveillance and had nonnegative containment delay were analyzed. For serial intervals, only settings that linked 2-4 cases, had identifiable infectors, and were related to local transmission were used to generate infector-infectee pairs for analysis. For R_t_, only symptomatic local cases were considered.

A Markov chain Monte Carlo with doubly interval-censored likelihood [16] was adopted to fit the empirical containment delay and serial interval with four candidate distributions (gamma, lognormal, weibull, and normal) with credible intervals computed. The smallest value of the leave-one-out cross-validation information criterion (LOOIC) [17] indicates the best fitted distribution. Assuming the best fitted distribution for serial intervals as the weighted infectivity function, incidence data of local cases by dates of symptom onset with an 8-day window (to reflect the time between exposure to SARS-CoV-2 and symptom onset) were fitted by a novel and statistically robust tool for estimating R_t_ [18].

Assuming the respective best fitted distributions as the likelihood function, multivariate linear regression models were used to examine the associated factors for containment delays and serial intervals. The effect of each factor was measured in terms of percentage contribution as previously described [19], which is defined as the percentage change in the predicted value of the outcome (from the regression models) relative to a comparator set of covariate values (referred to as “the comparator”). The comparator refers to a transmission event fulfilling these conditions: the case (for containment delay) or the infector (for serial interval) was female, aged 0-30 years, free of chronic diseases, free of general and respiratory symptoms; the transmission happened in a household, during wave 1, and is of the secondary generation; and (for serial interval only) the mobility index being the baseline value on the onset date of the infector. Specifically, ln(α_i_ + j + 1) and ln(β_i_ + k + 1) were defined as the response variables for the multivariable regression analyses, where α_i_ and β_i_ are the containment delay and serial interval of the i^th^ case, respectively, and j and k are the maximum absolute values for the nonpositive containment delays and the nonpositive serial intervals, respectively.

A statistical significance of .05 was specified. All analyses were performed in R (version 3.6.3; R Foundation for Statistical Computing) [20] with the RStan package (version 2.19.3).

### Ethical Statement

This study was approved by the Survey and Behavioral Research Ethics Committee of The Chinese University of Hong Kong (reference: SBRE-19-595).

### Data Statement

The availability of the data set is subject to approval from HKCHP and relevant government departments.

## Results

### Characteristics of the Two Epidemic Waves

**Figure 1:**
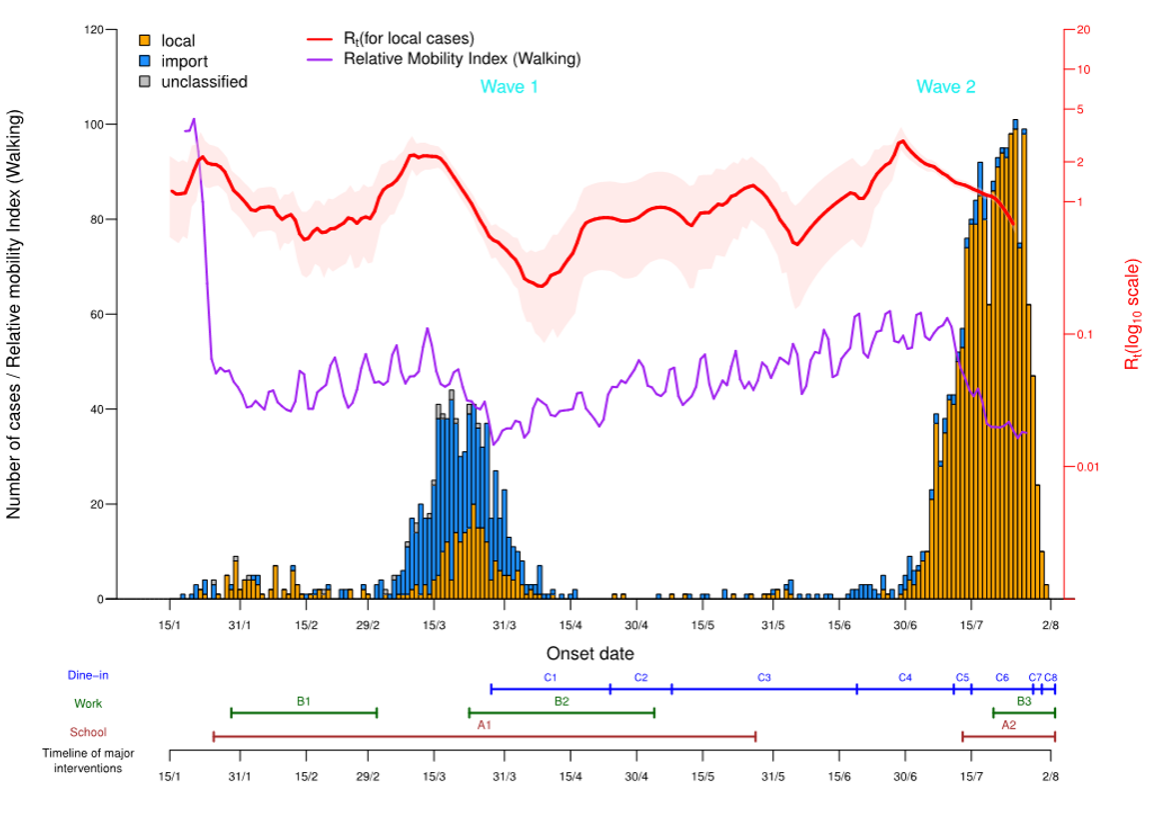
Epidemic curve of COVID-19 and timeline for major interventions in Hong Kong. A1: School closure, January 25-May 26, 2020. A2: School closure, July 13, 2020 to ongoing as of August 2, 2020. B1: The government spearheaded work-from-home arrangements, January 29-March 1, 2020. B2: The government spearheaded work-from-home arrangements, March 23-May 3, 2020. B3: The government spearheaded work-from-home arrangements, July 20, 2020 to ongoing as of August 2, 2020. C1: Regulations imposed on dine-in services, March 28-April 23, 2020: (1) tables ≥1.5 meters apart, (2) ≤4 persons per table, and (3) number of customers ≤50% of capacity. C2: Regulations imposed on dine-in services, April 24-May 7, 2020: (1) tables ≥1.5 meters apart and (2) ≤4 persons per table. C3: Regulations imposed on dine-in services, May 8-June 18, 2020: (1) tables ≥1.5 meters apart and (2) ≤8 persons per table. C4: Regulations imposed on dine-in services, June 19-July 10, 2020: tables ≥1.5 meters apart. C5: Regulations imposed on dine-in services, July 11-July 14, 2020: (1) tables ≥1.5 meters apart, (2) ≤8 persons per table, and (3) number of customers ≤60% of capacity. C6: Regulations imposed on dine-in services, July 15-July 28, 2020: (1) tables ≥1.5 meters apart, (2) ≤4 persons per table, (3) number of customers ≤50% of capacity, and (4) no dine-in service from 6 PM to 4:59 AM every day. C7: No dine-in service at any time, July 29-July 30, 2020. C8: Regulations imposed on dine-in services, July 31, 2020 to ongoing as of August 2, 2020: (1) tables ≥1.5 meters apart, (2) ≤2 persons per table, (3) number of customers ≤50% of capacity, and (4) no dine-in service from 6 PM to 4:59 AM every day.

The report date, June 14, 2020, defined two epidemic waves in Hong Kong: (1) January 23-June 14, 2020 (wave 1) and (2) June 15-August 2, 2020 (wave 2). This cut-off date separated the clusters in both waves without overlap. Two epochs were further defined within wave 1: (1) January 23-February 29, 2020 (epoch 1) and (2) March 1-June 14, 2020 (epoch 2). Wave 1 was initially dominated by imported cases from mainland China and local cases (epoch 1) and subsequently by imported cases from Europe and the Americas (epoch 2), whereas wave 2 was composed mainly of local cases (Figure 1).

The government of Hong Kong has enacted multipronged interventions, and the major ones include school closures, work-from-home arrangements, and limiting customer flow and time for dine-in services (Figure 1). In addition, the public voluntarily reduced their mobility substantially, such that the mobility index dropped from 100.2 initially to as low as 28.0 as the epidemic progressed (Figure 1).

R_t_ was generally below 1 throughout the epidemic, but it peaked at 2.39 in wave 1 and 3.04 in wave 2. This elevated R_t_, coupled with the short doubling time of the epidemic size in wave 2 (Figure S1 in Multimedia Appendix 1), suggested that Hong Kong was on the verge of an uncontrolled outbreak during wave 2.

### Characteristics of Cases

As of August 2, 2020, 3512 cases were reported. The mean age of cases was 43.91 (SD 20.27) years, with half of them being male (1748/3512). In addition, 6.2% (216/3512) had consulted with a doctor before case confirmation, and 9.7% (339/3512) had chronic conditions (Table S2 in Multimedia Appendix 1). Most cases (2649/3512, 75.4%) were symptomatic, with fever (929/2649, 35.1%) and cough (805/2649, 30.4%) being the most common symptoms (Table S3 in Multimedia Appendix 1). The proportion of asymptomatic cases decreased from the younger age groups to the older ones (0-9 years: 34.9%; 10-19 years: 34.9%; 20-29 years: 21.7%; 30-39 years: 22.4%; 40-49 years: 18.7%; 50-59 years: 16.1%; 60-69 years: 12.7%; 70-79 years: 21.7%; and ≥80 years: 12.9%). In terms of the probable source of infection, 67.4% (2366/3512) were locally acquired cases (Table 1), among whom 1575 cases were symptomatic and neither quarantined nor under medical surveillance (Table 2).

**Table 2:** Classification of 3512 cases in Hong Kong, as of August 2, 2020.

Case classification					Wave 1 (n=1110), n (%)		Wave 2 (n=2402), n (%)		Total (n=3512), n (%)
**Imported case**									
	Symptomatic			554 (49.9)		94 (3.9)		648 (18.5)	
	Asymptomatic			189 (17)		231 (9.6)		420 (12)	
	Missing			0 (0)		48 (2)		48 (1.4)	
**Local case**									
	**Symptomatic**								
		Quarantine^a^		31 (2.8)		158 (6.6)		189 (5.4)	
		**Nonquarantine**							
			Medical surveillance	91 (8.2)		125 (5.2)		216 (6.2)	
			Others^b^	176 (15.9)		1399 (58.2)		1575 (44.8)	
	Asymptomatic			39 (3.5)		246 (10.2)		285 (8.1)	
	Missing			0 (0)		101 (4.2)		101 (2.9)	
Unclassified					30 (2.7)		0 (0)		30 (0.9)

^a^This included home/hotel confinee cases, camp/center quarantine cases, and cases released from home/hotel quarantine before getting infected.

^b^This included cases with these modes of case detection: enhanced lab surveillance, enhanced surveillance in private, enhanced surveillance at general outpatient clinics and accident and emergency departments, meeting reporting criteria, diagnosed in private clinic, test at private clinic, and being under Tier 7 classification.

### Containment Delay

After excluding one case with negative containment delay, 1574 cases were included in the estimation. Log-normal distribution (mean 5.18 [SD 3.04] days) fitted the empirical containment delay best (LOOIC: 7525.6; Table S4 in Multimedia Appendix 1), which ranged from 4.38 days (95% empirical CI [eCI] 3.80-4.95) for cases belonging to the secondary settings or beyond in a cluster to 6.48 days (95% eCI 6.15-6.82) for cases identified through a private mode of detection (Table 3).

**Table 3:** Estimates of and factors associated with containment delay based on 1574 cases.

Factors			Cases, n (%)		Subgroup-specific estimates (95% empirical CI)		Percentage contribution (95% CI)
**Age group (years)**							
	0-30	301 (19.1)		4.87 (4.55, 5.18)		Reference^c^	
	31-45	358 (22.7)		4.86 (4.58, 5.14)		0.04 (–7.82, 8.48)	
	46-60	448 (28.5)		5.27 (4.99, 5.55)		6.86 (–1.14, 15.41)	
	≥61	467 (29.7)		5.52 (5.21, 5.82)		11.29 (3.05, 20.09)	
**Sex**							
	Female	834 (53)		5.15 (4.95, 5.35)		Reference^c^	
	Male	740 (47)		5.20 (4.98, 5.42)		2.11 (–3.17, 7.63)	
**Number of general symptoms**							
	0	985 (62.6)		5.38 (5.20, 5.56)		Reference^c^	
	≥1	589 (37.4)		4.83 (4.58, 5.08)		–12.45 (–17.39, –7.27)	
**Number of respiratory symptoms**							
	0	1002 (63.7)		5.17 (4.99, 5.35)		Reference^c^	
	1	459 (29.2)		5.20 (4.91, 5.49)		–1.25 (–7.09, 4.91)	
	≥2	113 (7.2)		5.08 (4.54, 5.62)		1.86 (–8.77, 13.57)	
**Order of settings**							
	None	423 (26.9)		5.71 (5.43, 6.00)		Reference^c^	
	Primary	1045 (66.4)		5.03 (4.85, 5.22)		–10.08 (–15.44, –4.43)	
	Secondary or beyond	106 (6.7)		4.38 (3.80, 4.95)		–20.73 (–29.61, –10.93)	
**Mode of case detection**							
	Private^a^	347 (22)		6.48 (6.15, 6.82)		Reference^c^	
	Public^b^	1227 (78)		4.80 (4.64, 4.96)		–27.56 (–32.33, –22.52)	
**Wave**							
	1	176 (11.2)		5.29 (4.78, 5.80)		Reference^c^	
	2	1398 (88.8)		5.16 (5.00, 5.31)		–7.23 (–15.58, 1.82)	

^a^Private mode includes diagnosis in private, enhanced surveillance in private, and private test.

^b^Public mode includes enhanced lab surveillance, enhanced surveillance at general outpatient clinics and accident and emergency departments, meeting reporting criteria, Tier 6, and Tier 7.

^c^The reference is a common comparator across all variables.

Containment delay varied by several factors (Table 3; Figure S2A in Multimedia Appendix 1). Notably, the percentage contribution (relative to the comparator) significantly increases with older age (≥61 years) by 11.29% (95% CI 3.05%-20.09%). On the contrary, the percentage contribution decreases with the following: (1) the presence of general symptoms (12.45%, 95% CI 7.27%-17.39%); (2) the transmission event originating from a primary setting of a cluster (10.08%, 95% CI 4.43%-15.44%); (3) the transmission event originating from a secondary setting or beyond of a cluster (20.73%, 95% CI 10.93%-29.61%); and (4) private mode of case detection (27.56%, 95% CI 22.52%-32.33%). The containment delay was shorter in wave 2 than in wave 1, though not statistically significant; the percentage contribution by wave 2 was –7.23% (95% CI –15.58% to 1.82%).

### Serial Interval

Initially, there were 847 settings linking at least two cases. After removing 144 settings consisting of solely imported cases, 72 settings with ≥5 cases (as the simultaneous presence of a large number of cases in a setting obscures the transmission link between cases), and 104 settings in which the infectors could not be identified (for example, there was no, or more than one, index case), 528 settings remained. From these 528 settings, 757 infector-infectee pairs were generated. After further removing 4 pairs with duplicated infectees and 195 pairs with missing onset dates of infectors/infectees, 558 paired data were included in the analysis. The mean number of infectees per infector was 1.46.

There were 17 negative serial intervals (range: –5 to –1 days). Log-normal distribution (mean 4.74 [SD 4.24] days) fitted the overall empirical serial intervals best (LOOIC: 3095.5; Table S4 in Multimedia Appendix 1). The serial intervals were 6.70 days (95% eCI 5.45-7.95) and 4.35 days (95% eCI 4.00-4.70) in waves 1 and 2, respectively. Further, the subgroup estimates of serial intervals ranged from 2.18 days (95% eCI –0.52 to 4.88) for quaternary transmission or beyond to 5.85 days (95% eCI 4.57-7.13) among the infector-infectee pairs with infectors with chronic conditions (Table 4).

**Table 4:** Estimates of and factors associated with serial interval based on 558 infector-infectee pairs.

Factors			Pairs, n (%)		Subgroup-specific estimates (95% empirical CI)		Percentage contribution (95% CI)
**Age group of infector (years)**							
	0-30	77 (13.8)		4.12 (3.41, 4.83)		Reference^c^	
	31-45	121 (21.7)		4.43 (3.53, 5.33)		–0.24 (–16.50, 17.88)	
	46-60	179 (32.1)		4.84 (4.26, 5.43)		10.47 (–6.17, 28.92)	
	≥61	181 (32.4)		5.12 (4.42, 5.82)		7.57 (–9.28, 26.33)	
**Sex of infector**							
	Female	295 (52.9)		4.83 (4.34, 5.33)		Reference^c^	
	Male	263 (47.1)		4.64 (4.10, 5.18)		–5.52 (–14.81, 4.37)	
**Presence of chronic conditions among infectors**							
	No	485 (86.9)		4.58 (4.20, 4.95)		Reference^c^	
	Yes	73 (13.1)		5.85 (4.57, 7.13)		0.41 (–15.17, 17.67)	
**Number of general symptoms presented by infectors**							
	0	338 (60.6)		4.78 (4.32, 5.25)		Reference^c^	
	≥1	220 (39.4)		4.69 (4.09, 5.28)		–6.34 (–16.48, 4.53)	
**Number of respiratory symptoms presented by infectors**							
	0	336 (60.2)		4.22 (3.83, 4.61)		Reference^c^	
	≥1	222 (39.8)		5.54 (4.84, 6.23)		8.29 (–3.07, 20.48)	
**Type of setting**							
	Household	390 (69.9)		4.83 (4.41, 5.25)		Reference^c^	
	Institution	10 (1.8)		5.00 (2.59, 7.41)		4.05 (–30.27, 47.56)	
	Social activity	113 (20.3)		4.27 (3.54, 4.99)		–9.17 (–20.78, 3.41)	
	Work	45 (8.1)		5.16 (3.12, 7.19)		–4.00 (–21.02, 15.12)	
**Wave**							
	1	94 (16.8)		6.70 (5.45, 7.95)		Reference^c^	
	2	464 (83.2)		4.35 (4.00, 4.70)		–33.90 (–45.08, –21.63)	
**Order of transmission**							
	Secondary	445 (79.7)		4.89 (4.49, 5.30)		Reference^c^	
	Tertiary	102 (18.3)		4.36 (3.46, 5.27)		–17.31 (–28.84, –4.75)	
	Quaternary or beyond	11 (2)		2.18 (–0.52, 4.88)		–50.75 (–72.53, –23.45)	
Relative mobility index^a,b,c^			N/A^d^		N/A		0.83 (0.32, 1.33)

^a^An 8-day lag was assumed to account for the time between exposure to SARS-CoV-2 and the first symptom onset, based on the estimated 7.76-day incubation period [21].

^b^The original mobility index was further adjusted relative to January 18, 2020, which has a value of 100.

^c^The reference is a common comparator across all variables.

^d^N/A: not applicable.

Serial intervals varied by several factors (Table 4; Figure S2B in Multimedia Appendix 1). They were significantly shorter in wave 2 than in wave 1: the percentage contribution by wave 2 was –33.9% (95% CI –45.08% to –21.63%). Serial intervals also decreased with a tertiary transmission (percentage contribution: –17.31%, 95% CI –28.84% to –4.75%), and a quaternary transmission or beyond (percentage contribution: –50.75%, 95% CI –72.53% to –23.45%). On the other hand, the 8-day lagged relative mobility index (assuming an incubation period of 7.76 days [21], which reflected the possible exposure date for each infector-infectee pair) lengthened the serial interval (percentage contribution: 0.83%, 95% CI 0.32%-1.33%).

### Transmission Events

**Figure 2:**
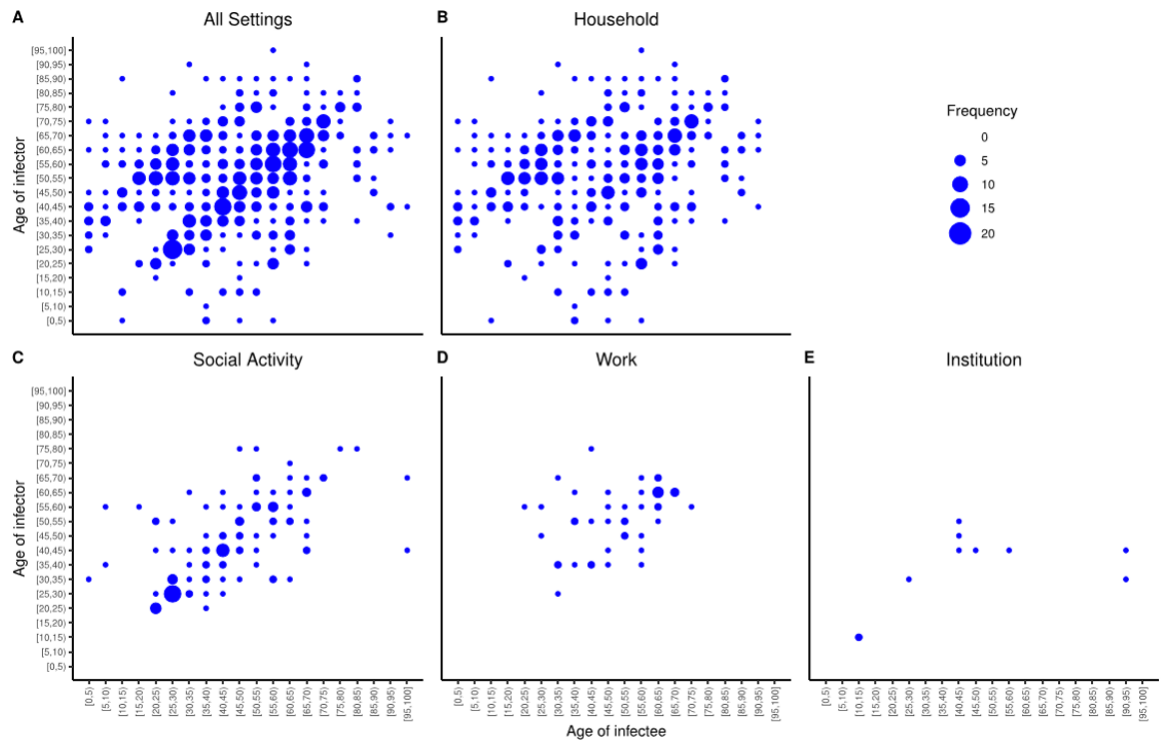
Age-specific transmission events in (A) all settings, (B) households, (C) social settings, (D) work settings, and (E) institutions.

Transmission events that occurred within the same age band were high (101/558, 18.1%) when using 5-year age bands (Figure 2A). Similar age transmission patterns were also observed in households (Figure 2B) and in social settings (Figure 2C), but were less obvious in work (Figure 2D) and institutional (Figure 2E) settings. In the household setting, transmission from infectors in older age groups (50-70 years) to infectees in younger age groups (15-40 years) was commonly observed (Figure 2B). The age transmission matrix asymmetry in all settings well reflected this phenomenon as well as the lack of transmission from younger age groups to older ones (Figure 2A).

Importantly, households (390/558, 69.9%) and social settings (113/558, 20.3%) were the two most common settings for transmission in Hong Kong (Table 4) in both epidemic waves (Table S5 in Multimedia Appendix 1).

## Discussion

### Summary of Study Findings

Understanding the evolving epidemiology of an infectious disease is vital for guiding infection control policies. From the case series between January 23, 2020, and August 2, 2020, we identified two waves of epidemics. R_t_ was in general below the outbreak threshold, suggesting that interventions, whether adopted voluntarily by the community [22] or institutionalized by the government [8], successfully interrupted the transmission of COVID-19. As the epidemic grew in Hong Kong, containment delays and serial intervals were shortened over time. The shortening of the former was associated with the manifestation of general symptoms, involvement in clusters, and public mode of case detection, whereas that of the latter was associated with later transmission generations and lower mobility. The occurrence of intra–age group transmission is more common than inter–age group transmission, and households and social settings together account for 90.14% of all identified transmission events.

### Result Implications

Our results have five implications. First, the factors associated with a reduction in containment delay suggested that government-enacted interventions were useful in achieving COVID-19 outbreak control in Hong Kong and should be further encouraged. Containment delay played a major role in determining whether an outbreak was controllable; assuming 80% of contacts can be traced, the chance of controlling an outbreak fell from 89% to 31% if the containment delay increased from 3.43 days to 8.09 days [23]. The difference in containment delay experienced by cases detected by different modes (private: 6.48 days; public: 4.80 days) pinpointed the worthiness of continual investment in public modes of case detection, including setting up community testing centers and mobile specimen collection stations. Further, as reflected by the percentage contribution by cases involved in a cluster (–20.73% to –10.08%), contact tracing and the follow-up quarantine of individuals with epidemiological links with cases were useful in reducing containment delay; contact tracing should involve a high tracing ratio (preferably ≥80%) and should be done when there are only a few initial cases [23].

Second, the association between decreasing serial intervals over time and lower mobility aligned with the contention that serial intervals are shortened by nonpharmaceutical interventions [11]. Reduced serial intervals indicated faster case generation replacement, which may be attributable to the institutionalized social-distancing policies (Figure 1) that diminished the geographical reach of citizens. This is a tradeoff between time spent in a place versus number of places visited in a limited time, such that citizens may stay in confined locations (eg, home) longer. Although the underlying mechanisms remain undetermined, we hypothesize that confined geographical movement would, in reality, intensify the proximity of contacts between successive case generations. This hypothesis is in line with earlier findings that more time spent in close proximity to the index case shortened serial intervals of influenza [10]. Although the adopted mobility index remained composite, empirical contact surveys—such as the ones by Mossong and colleagues [24] and Kwok and colleagues [19]—that disentangle the interplay of contact dimensions will advance research in the area of evolving epidemiology. On one hand, it is of interest to dissect the role of different contact dimensions on disease transmission; on the other, the potential of other composite social mobility measures—such as the Twitter Social Mobility Index [25]—in estimating epidemiological parameters should be explored.

Third, the presence of negative serial intervals, suggestive of presymptomatic transmission and asymptomatic cases, is a reminder for the community to stay on guard against the resurgence of COVID-19. Infections happening before symptom onset would impede the effectiveness of control measures. This presymptomatic fraction appeared to be low in this study (17/558; 3.05%), but it can be as high as 12.6% [26]. Meanwhile, the proportion of asymptomatic local cases was 12.0% (285/2366) and the number of accumulated infections so far (ie, 3512 cases among more than 7 million people in Hong Kong) is not high enough to confer herd immunity when compared with the conservative 5.66% previously suggested [27]. Together, coupled with the fact that vaccinations will take some time, these results suggest that the community should remain vigilant against any resurgence.

Fourth, temporal variations of key epidemiological parameters should be considered. It is common to assume that the empirical data (of the epidemiological parameters) resembles theoretical distributions. However, the correlation of lower mobility (as proxy to voluntary or compulsory social distancing en masse), involvement in local clusters (as proxy to early government actions on case tracing), and government-level case identification with either containment delay or serial interval observed in this study suggests that epidemiological parameters are dynamic throughout the epidemic. Furthermore, with SARS-CoV-2 transcending international borders, it has mutated into different clusters or subtypes [28-30]. These subtypes differ by their intrinsic properties, exhibiting variations in COVID-19 epidemiology [30]. Therefore, caution must be taken to interpret findings from infectious disease models that assume static epidemiological parameters.

Fifth, the high proportion of transmission events within the same age group supports the ban on gatherings outside of households. The observed intra–age group transmission of COVID-19 echoed the contact assortativity by age of the Hong Kong population [19], and is in line with earlier research [31]: social contact should be considered together with age when it comes to determining the driving force of the incidence of respiratory infections. Further, the asymmetric age transmission matrix revealed that children rarely infected others in the first two epidemic waves. This phenomenon may be attributable to the continual school closure, resulting in fewer social interactions than usual among children in Hong Kong. With the reopening of schools, and hence more social mixing among children, transmission chains branching from children are possible; therefore, older adults who are frequently in contact with children should be prioritized to receive the COVID-19 vaccine, as older adults have a higher risk of COVID-19–related mortality [32]. In addition, the abundance of transmission events in households underscores the need for residence-centered preventive measures, as per the lesson learned from aerosol transmission (through defective plumbing) of the 2003 severe acute respiratory syndrome virus in the Amoy Gardens housing complex in Hong Kong [33].

### Study Limitations

There are two study limitations that bear mentioning. First, the data in this study, including self-reported symptoms and contact history, were subject to recall bias. However, the medical surveillance in place to monitor the contacts of cases may lessen the data uncertainties. Second, some cases might not have been captured by the present surveillance system in Hong Kong due to the underdiagnosis of mild cases and asymptomatic individuals, who might play a role in the transmission chain involving unlinked local cases.

## Appendix

Multimedia Appendix 1 Supplementary data.

## Abbreviations

eCI: empirical confidence interval
HKCHP: Hong Kong Centre for Health Protection
LOOIC: leave-one-out cross-validation information criterion
