# Supplementary material for: Evolving Epidemiological Characteristics of COVID-19 in Hong Kong From January to August 2020: Retrospective Study
Source: J Med Internet Res. 2021 Apr 16;23(4):e26645. doi: 10.2196/26645 (PMC8054773; doi:10.2196/26645)
Supplement: Multimedia Appendix 1 [file jmir_v23i4e26645_app1.docx]

**Multimedia Appendix 1**

Table S1. Grouping of symptoms

| **Classification** |  | **Symptoms ^a^** |
| --- | --- | --- |
| General |  | fever, myalgia, malaise, chills, headache, fatigue, dizziness, dry mouth, bone pain ^b^, poor appetite, sweating, heaviness over head, joint pain ^b^, rigors, decrease general condition (GC), weakness, sore itchiness |
| Circulatory and respiratory systems |  | cough, blocked nose ^b^, shortness of breath (SOB), runny nose ^b^, throat itchiness, dry throat, throat discomfort, sore throat, irritable throat, sputum, chest pain, chest discomfort, sneezing, fast atrial fibrillation (AF), influenza-like illness (ILI). |
| Digestive system and abdomen |  | Diarrhea ^b^, vomiting, epigastric pain, nausea, abdominal pain. |
| Cognition, perception, emotional state and behaviour |  | decreased sensation of taste, decreased sensation of smell, loss of taste, loss of smell, loss of consciousness, drowsiness. |
| Musculoskeletal system |  | back pain ^b^ |
| Urinary system |  | acute renal failure, decreased urine output |
| Skin and subcutaneous tissue |  | lower limb swelling |
| Eyes |  | conjunctivitis^­­ b^ |
| Others |  | sepsis, desaturation, AED, delirium tremens (DTS), spectum |
| ^a^ The classification is based on ICD-10 unless specified. | | |
| ^b^ The classification is based on “The National Ambulatory Medical Care Survey: Symptom Classification”. | | |

Table S2. Frequency of chronic diseases among the 339 cases with chronic conditions

| **Chronic disease** | **N (%)** |  | **Chronic disease** | **N (%)** |
| --- | --- | --- | --- | --- |
| Hypertension | 84 (24.8) |  | Bed ridden | 1 (0.3) |
| Diabetes | 52 (15.3) |  | CA corpus | 1 (0.3) |
| Hyperlipidaemia | 24 (7.1) |  | CA kidney | 1 (0.3) |
| Heart disease ^a^ | 18 (5.3) |  | CA liver | 1 (0.3) |
| Liver disease ^b^ | 17 (5.0) |  | CA pancreas | 1 (0.3) |
| Asthma | 14 (4.1) |  | Colonic tubular adenoma | 1 (0.3) |
| Thyroid disease | 12 (3.5) |  | COPD | 1 (0.3) |
| Renal disease^c^ | 9 (2.7) |  | Diverticulosis | 1 (0.3) |
| Depression | 8 (2.4) |  | Emphysema | 1 (0.3) |
| Gout | 8 (2.4) |  | Gallbladder stone | 1 (0.3) |
| Hypercholesterolaemia | 6 (1.8) |  | Gallstone | 1 (0.3) |
| Stroke ^d^ | 6 (1.8) |  | Gastroduodenal ulcer | 1 (0.3) |
| Anaemia | 5 (1.5) |  | GERD | 1 (0.3) |
| Dyslipidaemia ^e^ | 5 (1.5) |  | Hip and Spine problem | 1 (0.3) |
| BPH | 4 (1.2) |  | Jugular venous thrombosis | 1 (0.3) |
| CA breast | 4 (1.2) |  | Laparoscopic Cholecystectomy | 1 (0.3) |
| Dementia | 4 (1.2) |  | Left vestibular schwannoma with brainstem compression | 1 (0.3) |
| CA prostate | 3 (0.9) |  | Leukemia | 1 (0.3) |
| Epilepsy | 3 (0.9) |  | Lyme disease | 1 (0.3) |
| HIV | 3 (0.9) |  | Lymphoma | 1 (0.3) |
| Impaired fasting glucose | 3 (0.9) |  | Multiple myeloma | 1 (0.3) |
| Obesity | 3 (0.9) |  | Oncology FU | 1 (0.3) |
| PD ^f^ | 3 (0.9) |  | Osteoarthritis of knee | 1 (0.3) |
| Sleep apnoea | 3 (0.9) |  | Peripheral vascular disease | 1 (0.3) |
| Allergic rhinitis | 2 (0.6) |  | Primary amenorrhoea | 1 (0.3) |
| Anxiety | 2 (0.6) |  | Prostatism | 1 (0.3) |
| Arrhythmia | 2 (0.6) |  | Psoriatic arthritis | 1 (0.3) |
| CA lung | 2 (0.6) |  | PTSD | 1 (0.3) |
| Gastric disease ^g^ | 2 (0.6) |  | Rectal Cancer | 1 (0.3) |
| Lung nodule | 2 (0.6) |  | Rheumatoid arthritis | 1 (0.3) |
| Mental retardation | 2 (0.6) |  | Schizophrenia | 1 (0.3) |
| Old TB | 2 (0.6) |  | Sleep disorder | 1 (0.3) |
| Osteoporosis | 2 (0.6) |  | Trigeminal neuralgia | 1 (0.3) |
| Psychosis | 2 (0.6) |  | Ureteric stone | 1 (0.3) |
| Urinary tract problem | 2 (0.6) |  | Zoster | 1 (0.3) |
| ^a^Include complete heart block, IHD (ischemic heart disease), cardiac arrhythmia, MR (mitral regurgitation), TOF (Tetralogy of Fallot). | | | | |
| ^b^Include fatty liver, liver transplantation, Hep B (Hepatitis B). | | | | |
| ^c^Include chronic renal disease, ESRF (end stage renal disease), renal stone, renal transplantation. | | | | |
| ^d^Include CVA (cerebrovascular accident),TIA(transient ischemic attack). | | | | |
| ^e^Include lipid | | | | |
| ^f^Include Parkinsonism. | | | | |
| ^g^Include chronic gastritis, gastric ulcer. | | | | |

Abbreviations: CA, cancer; BPH, benign prostatic hyperplasia; PD, Parkinson’s disease; HIV, human immunodeficiency virus; TB, Tuberculosis; COPD, Chronic obstructive pulmonary disease; GERD, Gastroesophageal Reflux Disease; PTSD, [Post-traumatic stress disorder.](https://www.mayoclinic.org/diseases-conditions/post-traumatic-stress-disorder/symptoms-causes/syc-20355967)

Table S3. Frequency of symptoms among the 2649 symptomatic cases

| **Symptom** | **N (%)** |  | **Symptom** | **N (%)** |
| --- | --- | --- | --- | --- |
| Fever^a^ | 929 (35.1) |  | Chest discomfort | 2 (0.1) |
| Cough^b^ | 805 (30.4) |  | Decreased sensation of taste | 2 (0.1) |
| Sore throat | 353 (13.3) |  | ILI | 2 (0.1) |
| Runny nose | 180 (6.8) |  | Joint pain | 2 (0.1) |
| Headache^c^ | 178 (6.7) |  | Acute renal failure | 1 (0.0) |
| Shortness of breath^d^ | 88 (3.3) |  | AED | 1 (0.0) |
| Loss of smell | 84 (3.2) |  | Conjunctivitis | 1 (0.0) |
| Diarrhea | 80 (3.0) |  | Decrease GC | 1 (0.0) |
| Myalgia^e^ | 61 (2.3) |  | Decreased sensation of smell | 1 (0.0) |
| Loss of taste | 50 (1.9) |  | Decreased urine output | 1 (0.0) |
| Malaise | 45 (1.7) |  | Desaturation | 1 (0.0) |
| Chills | 39 (1.5) |  | Drowsiness | 1 (0.0) |
| Blocked nose^f^ | 28 (1.1) |  | DTS | 1 (0.0) |
| Throat discomfort | 27 (1.0) |  | Epigastric pain | 1 (0.0) |
| Sputum | 25 (0.9) |  | Fast AF | 1 (0.0) |
| Fatigue^g^ | 22 (0.8) |  | Heaviness over head | 1 (0.0) |
| Throat itchiness | 17 (0.6) |  | Irritable throat | 1 (0.0) |
| Dizziness | 15 (0.6) |  | Loss of consciousness | 1 (0.0) |
| Chest pain | 11 (0.4) |  | Lower limb swelling | 1 (0.0) |
| Dry throat | 11 (0.4) |  | Rigors | 1 (0.0) |
| Vomiting | 11 (0.4) |  | Sepsis | 1 (0.0) |
| Poor appetite^h^ | 6 (0.2) |  | Sneezing | 1 (0.0) |
| Abdominal pain | 5 (0.2) |  | Sore itchiness | 1 (0.0) |
| Back pain | 4 (0.2) |  | Spectum | 1 (0.0) |
| Nausea | 4 (0.2) |  | Sweating | 1 (0.0) |
| Dry mouth | 3 (0.1) |  | Weakness | 1 (0.0) |
| Bone pain | 2 (0.1) |  |  |  |
| ^a^Include “Fver”. | | | | |
| ^b^Include “ough” | | | | |
| ^c^Include “Headhche” | | | | |
| ^d^Include “shortness and breath” | | | | |
| ^e^Include muscle pain. | | | | |
| ^f^Include stuffy nose | | | | |
| ^g^include tired | | | | |
| ^h^include loss of appetite. | | | | |

Abbreviations: ILI, Influenza-like illness; GC, general condition; AF, atrial fibrillation; DTS, delirium tremens.

Table S4. Non-truncated and bootstrapped estimates for containment delay and serial interval

|  |  |  | **Gamma** | **Log-normal** | **Weibull** | **Normal** | **Bootstrap** |  | **Gamma** | **Log-normal** | **Weibull** | **Normal** | **Bootstrap** |  | **Gamma** | **Log-normal** | **Weibull** | **Normal** | **Bootstrap** |
| --- | --- | --- | --- | --- | --- | --- | --- | --- | --- | --- | --- | --- | --- | --- | --- | --- | --- | --- | --- |
|  |  |  | **Wave 1 (176 cases)** | | | | |  | **Wave 2 (1398 cases)^a^** | | | | |  | **Total (1574 cases)^a^** | | | | |
| Containment Delay | Mean |  | **5.31**  **(4.81,5.87)** | 5.34  (4.78,5.99) | 5.33  (4.81,5.88) | 5.28  (4.78,5.79) | 5.29  (4.81,5.81) |  | 5.16  (5.01,5.31) | **5.16**  **(5.01,5.31)** | 5.17  (5.02,5.32) | 5.16  (5.01,5.32) | 5.16  (5.00,5.31) |  | 5.17  (5.03,5.32) | **5.18**  **(5.04,5.33)** | 5.18  (5.03,5.34) | 5.17  (5.02,5.32) | 5.17  (5.03,5.33) |
|  | SD |  | **3.51**  **(3.07,4.02)** | 4.03  (3.35,4.90) | 3.41  (3.06,3.83) | 3.45  (3.10,3.85) | 3.45  (3.06,3.85) |  | 2.75  (2.63,2.87) | **2.91**  **(2.76,3.08)** | 2.96  (2.86,3.06) | 2.93  (2.82,3.05) | 2.95  (2.75,3.15) |  | 2.83  (2.72,2.95) | **3.04**  **(2.88,3.20)** | 3.00  (2.90,3.11) | 2.98  (2.88,3.09) | 3.01  (2.83,3.21) |
|  | LOOIC |  | **906.8** | 912.4 | 910.2 | 938.6 | Nil |  | 6636.3 | **6594.2** | 6804.4 | 6998.4 | Nil |  | 7554.6 | **7525.6** | 7716.8 | 7940.9 | Nil |
|  |  |  | **Wave 1 (94 pairs)** | | | | |  | **Wave 2 (464 pairs)** | | | | |  | **Total (558 pairs)** | | | | |
| Serial Interval | Mean |  | 6.73  (5.70,7.90) | **6.52**  **(5.50,7.63)** | 6.80  (5.58,8.14) | 6.52  (5.29,7.75) | 6.70  (5.55,8.03) |  | **4.35**  **(4.01,4.70)** | 4.37  (4.02,4.74) | 4.33  (3.97,4.70) | 4.33  (3.99,4.68) | 4.35  (4.01,4.71) |  | 4.75  (4.42,5.09) | **4.74**  **(4.38,5.11)** | 4.72  (4.34,5.11) | 4.73  (4.36,5.09) | 4.74  (4.41,5.12) |
|  | SD |  | 5.27  (4.47,6.26) | **5.18**  **(4.31,6.38)** | 6.22  (5.50,7.16) | 6.11  (5.33,7.05) | 6.12  (4.25,7.74) |  | **3.74**  **(3.49,4.03)** | 3.97  (3.64,4.34) | 4.01  (3.80,4.25) | 3.83  (3.59,4.08) | 3.84  (3.45,4.24) |  | 4.07  (3.81,4.36) | **4.24**  **(3.91,4.60)** | 4.62  (4.39,4.86) | 4.38  (4.13,4.64) | 4.39  (3.85,4.99) |
|  | LOOIC |  | 571.9 | **559.1** | 596.1 | 613.9 | Nil |  | **2510.9** | 2520.8 | 2562.2 | 2568.6 | Nil |  | 3105.4 | **3095.5** | 3208.5 | 3241.1 | Nil |

^a^ One case with negative containment delay was excluded.

Table S5. Number of infector-infectee pairs included in the serial interval analysis, stratified by order of transmissions and settings

| **Order of transmission**  **by settings** |  | **Number of pairs** | | | | |
| --- | --- | --- | --- | --- | --- | --- |
|  |  | **Wave 1**  **(n=94) (%)** |  | **Wave 2**  **(n=464) (%)** |  | **Total**  **(n=558) (%)** |
| **Secondary** |  |  |  |  |  |  |
| Household ^a^ |  | 30 (31.9) |  | 290 (62.5) |  | 320 (57.3) |
| Work ^b^ |  | 4 (4.3) |  | 30 (6.5) |  | 34 (6.1) |
| Social activity ^c^ |  | 16 (17.0) |  | 67 (14.4) |  | 83 (14.9) |
| Institution ^d^ |  | 1 (1.1) |  | 7 (1.5) |  | 8 (1.4) |
| **Tertiary** |  |  |  |  |  |  |
| Household ^a^ |  | 22 (23.4) |  | 43 (9.3) |  | 65 (11.6) |
| Work ^b^ |  | 4 (4.3) |  | 5 (1.1) |  | 9 (1.6) |
| Social activity ^c^ |  | 12 (12.8) |  | 14 (3.0) |  | 26 (4.7) |
| Institution ^d^ |  | 1 (1.1) |  | 1 (0.2) |  | 2 (0.4) |
| **Quaternary** |  |  |  |  |  |  |
| Household ^a^ |  | 2 (2.1) |  | 3 (0.6) |  | 5 (0.9) |
| Work ^b^ |  | 0 (0.0) |  | 2 (0.4) |  | 2 (0.4) |
| Social activity ^c^ |  | 1 (1.1) |  | 2 (0.4) |  | 3 (0.5) |
| **Quinary** |  |  |  |  |  |  |
| Social activity ^c^ |  | 1 (1.1) |  | 0 (0.0) |  | 1 (0.2) |
| ^a^ It included family and roommate. | | | | | | |
| ^b^ It included concrete driver, Kerry Logistics, sheltered workshop, cafedecoral colleague. | | | | | | |
| ^c^ It included friend, partner, neighbor, dating, meal, banquet, dinner, restaurant (KinWing, CapitalCanton in MeiFoo, Outback in Tsim Sha Tsui, Fung Ying Seen Koon), bar (ChinaBar, Kagesha),coffee, shopping center (Grand Plaza), gym, mahjong and majoring, tutorial, transportation (bus and taxi), travel, birthday party, beautyparlour, chatting ,hugging and contact. | | | | | | |
| ^d^ It included School, RCHE, hospital/clinic (ambulance, nosocomial infection, eye clinic, GP consultation). | | | | | | |


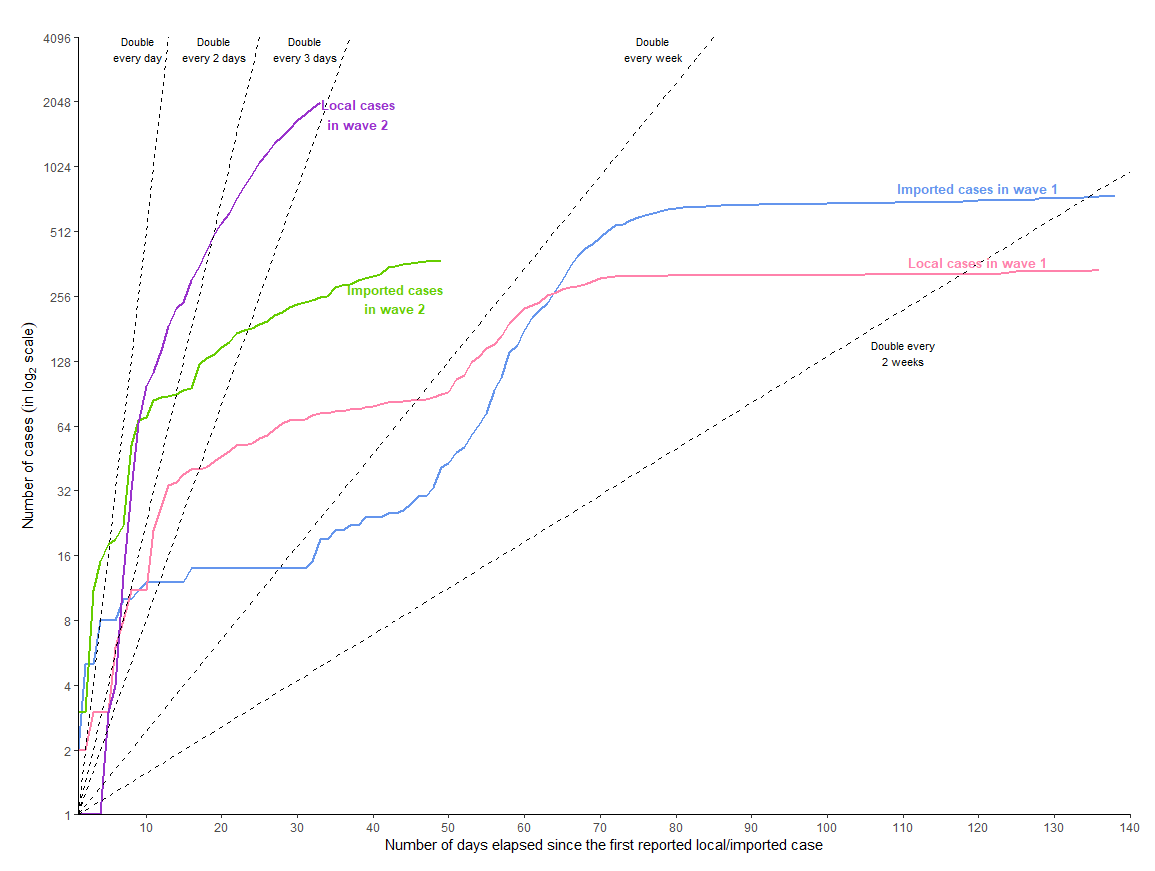


Figure S1. Cumulative number of COVID-19 cases by number of days elapsed since the first reported case(s) in two epidemic waves
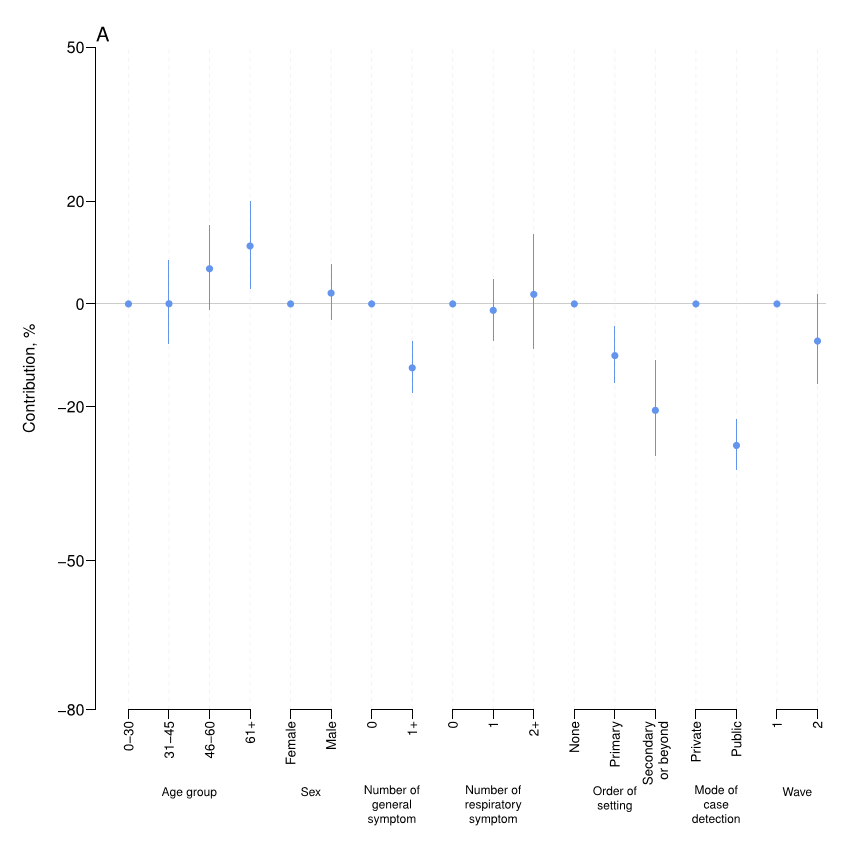

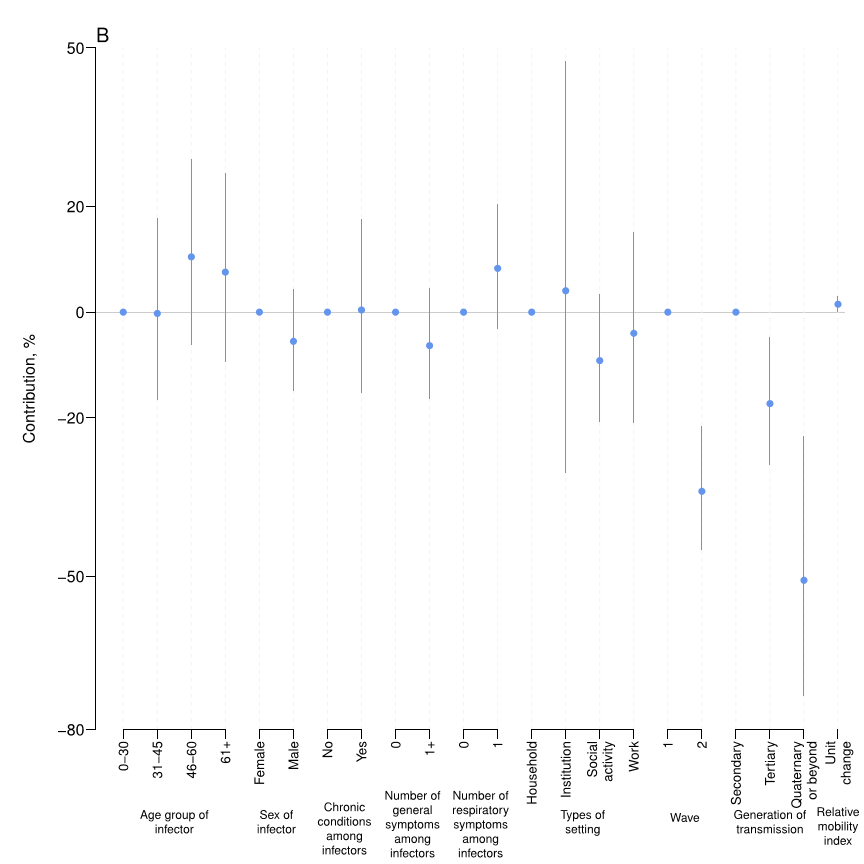


Figure S2. Percentage contribution of covariates in changing the length of (A) containment delay; and (B) serial interval.
